# Comparing single and multiple imputation strategies for harmonizing substance use data across HIV-related cohort studies

**DOI:** 10.1186/s12874-022-01554-4

**Published:** 2022-04-03

**Authors:** Marjan Javanbakht, Johnny Lin, Amy Ragsdale, Soyeon Kim, Suzanne Siminski, Pamina Gorbach

**Affiliations:** 1 Department of Epidemiology, Fielding School of Public Health, University of California, Los Angeles, Los Angeles, CA USA; 2 Statistical Methods and Data Analytics, Office of Advanced Research Computing, University of California, Los Angeles, Los Angeles, CA USA; 3 Frontier Science Foundation, Brookline, MA USA

## Abstract

**Background:**

Although standardized measures to assess substance use are available, most studies use variations of these measures making it challenging to harmonize data across studies. The aim of this study was to evaluate the performance of different strategies to impute missing substance use data that may result as part of data harmonization procedures.

**Methods:**

We used self-reported substance use data collected between August 2014 and June 2019 from 528 participants with 2,389 study visits in a cohort study of substance use and HIV. We selected a low (heroin), medium (methamphetamine), and high (cannabis) prevalence drug and set 10–50% of each substance to missing. The data amputation mimicked missingness that results from harmonization of disparate measures. We conducted Monte Carlo simulations to evaluate the comparative performance of single and multiple imputation (MI) methods using the relative mean bias, root mean square error (RMSE), and coverage probability of the 95% confidence interval for each imputed estimate.

**Results:**

Without imputation (i.e., listwise deletion), estimates of substance use were biased, especially for low prevalence outcomes such as heroin. For instance, even when 10% of data were missing, the complete case analysis underestimated the prevalence of heroin by 33%. MI, even with as few as five imputations produced the least biased estimates, however, for a high prevalence outcome such as cannabis with low to moderate missingness, performance of single imputation strategies improved. For instance, in the case of cannabis, with 10% missingness, single imputation with regression performed just as well as multiple imputation resulting in minimal bias (relative mean bias of 0.06% and 0.07% respectively) and comparable performance (RMSE = 0.0102 for both and coverage of 95.8% and 96.2% respectively).

**Conclusion:**

Our results from imputation of missing substance use data resulting from data harmonization indicate that MI provided the best performance across a range of conditions. Additionally, single imputation for substance use data performed comparably under scenarios where the prevalence of the outcome was high and missingness was low. These findings provide a practical application for the evaluation of several imputation strategies and helps to address missing data problem when combining data from individual studies.

## Introduction

Large-scale prospective cohort studies of those at risk for or living with HIV have been instrumental in investigating research questions that could not otherwise be accomplished through smaller studies. A number of cohorts have been established going as far back as the start of the HIV epidemic in the mid-1980s [1–6]. Some of the cohorts such as the multicenter AIDS cohort study (MACS) were set up as a single study across multiple sites, implementing the same protocol with standard data collection tools, while other studies such as the North American AIDS Cohort Collaboration on Research and Design (NA-ACCORD) were designed as a collaborative in which 25 cohorts collect and integrate a common set of core information [1, 3]. Smaller cohorts have an important role in addressing questions in sentinel populations. In the absence of a common data collection effort such as those in MACS and NA-ACCORD, strategies that allow us to compile data across these individual studies can help us achieve comparable effects, increasing the impact of the data collected.

The Collaborating Consortium of Cohorts Producing NIDA Opportunities (C3PNO) was established in 2017 by the National Institutes of Health/National Institutes of Drug Abuse (NIH/NIDA) to stimulate the use of NIDA longitudinal cohorts and to address high priority research on HIV/AIDS in the context of substance use (www.C3PNO.org). This consortium includes nine different cohorts located in the United States and Canada. All cohorts were established before the consortium was established, with the oldest having started in 1988 and the newest in 2015 [7, 8]. All cohorts focus on HIV and substance use, however the target population, participant sampling strategies, as well as data collection tools differs for each of these cohorts. Some of the cohorts are community-based, while others specifically focus on clinical populations. Furthermore, some cohorts are focused on young men who have sex with men (MSM) with others focused on persons who inject drugs. In order to allow for cross-cohort analyses, we implemented a rigorous data harmonization process for a core set of data elements. The specifics of the process have been described elsewhere [9], but briefly, we requested data dictionaries from each of the cohorts and identified a core set of variables including sociodemographic factors, clinical characteristics, and substance using behaviors. Common data elements were first reviewed by the consortium data team both qualitatively and quantitatively and the subsequent harmonized data sets were further reviewed with each cohort data team in order to ensure fidelity in the harmonization process. Given the consortium’s focus on substance use, we were particularly interested in maintaining as much information and specificity as possible related to substance use. While standardized measures of substance use were utilized by each cohort, the choice of measures differed across cohorts. Even when measures overlapped, most studies used variations making it challenging to harmonize data across studies. For instance, substance use was assessed with various time frames, including 30-day, 3-month, and 6-month recall periods. Combining these data to obtain substance use in the past six months could lead to misclassification bias particularly for occasional users who may not have used a given drug in the shorter recall periods.

This challenge to harmonizing substance use data – a key variable for the consortium – resulted in a patchwork pattern of missing data. There are a number of strategies to deal with missing data resulting from the harmonization processes where disparate measures cannot be collapsed into one variable. One common strategy is to ignore the missingness and use only participants with complete data in the analyses, which is well known for its potential for bias and inefficiency. A strategy to overcome this issue, which is widely used when dealing with missing data is imputation (i.e., replacing unknown or missing values with an estimate) then analyzing the full data set as if imputed values were observed. In recent years, as a result of significant advances in computing power, a wide array of techniques for producing imputations has emerged including regression based techniques that allow for specification of multivariable models, hot-deck techniques, as well as multiple imputation methods [10–12]. Additionally, strategies to evaluate the statistical properties of imputation techniques have also been explored, though few studies have taken a more applied and translational approach [13–15]. The objective of this study was to move beyond consideration of the statistical properties of these methods and present an applied overview of the performance of different imputation strategies when used for data harmonization. We used data from one of the cohorts participating in the consortium as a validation set and created missing data in such a way as to mimic the missingness that results during the harmonization process. We then applied three imputation strategies that vary in complexity including logistic regression, single hot-deck imputation, and multiple imputation and evaluated the performance of each strategy.

## Methods

### Data source

Data for this analysis were based on those collected from participants in the mSTUDY – one of the nine participating cohorts in the C3PNO consortium. The mSTUDY – an NIH/NIDA funded longitudinal study designed to assess the epidemiological and immunological impact of substance use and HIV on MSM – started study enrollment in August 2014 (and is ongoing) [16, 17]. Participants were recruited from two different study sites in Los Angeles, CA: a community-based organization providing services for the lesbian, gay, bisexual, and transgender community and a community-based university research clinic. Participants are eligible for mSTUDY if they are: (1) age 18 to 45 years at enrollment; (2) male at birth; (3) if HIV-negative, reported condomless anal intercourse with a male partner in the past 6-months; and (4) capable of providing informed consent. By design, half of the participants were people living with HIV. As well, half of the participants were substance users (self-report confirmed by urine drug screen).

### Data collection and substance use measures

At baseline and subsequent follow-up visits, which occurred at least six months apart, participants completed a self-administered, computer-based questionnaire. The questionnaire included questions on a number of domains ranging from sociodemographic characteristics, sexual risk behaviors, as well as an extensive battery of questions related to substance use. In this analysis we used substance use data collected as part of a modified version of the Alcohol, Smoking and Substance Involvement Screening Test (ASSIST) [18]. Specifically, for each substance participants were asked how often they have used it in the past six months. Substances of interest were cocaine, crack, ecstasy, heroin, cannabis, methamphetamine, poppers, and prescription drugs. Response options included never, once, monthly, weekly, and daily/almost daily. For the purpose of this analysis, all those who reported using a given substance at least once in the past six months were categorized as having used the particular drug, with all others being categorized as non-users. We selected drugs which were reported at low, medium, and high prevalence of use including heroin (prevalence 3%), methamphetamine (prevalence 38%), and cannabis (prevalence 52%), respectively. This allowed us to evaluate the performance of the imputation strategies under various prevalence estimates of the outcome.

### Analytic strategy

Data collected from August 2014 through June 2019, from 528 participants and the resulting 2,389 study visits were used in this analysis. A Monte Carlo simulation study with 500 iterations was run to assess the relative performance of each imputation method. At each iteration, first a proportion of the data was set to missing (i.e., data amputation) [19] with this step intended to mimic the missingness that results when we attempt to harmonize disparate measures across studies that measure substance use. Second, using the amputated data, three strategies including logistic regression scoring, single hot-deck, and multiple imputation were used to impute the missing data. Each imputation generated an estimated prevalence and confidence interval which was stored until 500 iterations were achieved. Finally, summary statistics across the 500 iterations allowed us to compare the performance of each strategy against the prevalence from the original data. Details of each of the steps in the process are described below.

#### Data amputation

Data amputation – the process of generating the missing data – involved simulations such that the original dataset (n = 2,389) was sampled with replacement (bootstrapped) and amputated giving consideration to several factors including the missing data mechanism, the amount of missing data, as well as the pattern of missingness [20]. The primary consideration for the missing data mechanism was whether the missingness was related to the underlying value for that variable. This is relevant given that strategies to handle missing data are largely reliant on correct assumptions of the mechanisms which caused the missingness [21]. For the purpose of this analysis we gave consideration to three different missing data mechanisms including missing completely at random (MCAR), missing at random (MAR), and missing not at random (MNAR) [22]. MCAR indicates there is no relationship between the missing data and any observed or unobserved variables. In this scenario, the probability of missing is the same for all cases in a given data set. MAR indicates a missing data mechanism in which there is a systematic relationship between the probability of missing and some observed data, but not the missing data itself. More specifically, under MAR the missingness is conditionally independent of *unobserved* outcomes (i.e. the missing data) but there is dependence on *observed* outcomes (i.e., auxiliary variables). The premise of MAR is that once the analyst controls for these auxiliary variables, the missingness is ignorable. Finally, MNAR suggests that there is a relationship between missingness and unobserved outcomes (i.e., the missing data), which makes it the most difficult mechanism to handle properly.

The level of missingness used in the amputation was set at 10, 30, and 50% in order to assess low to high rates of missing data. Additionally, the pattern of missingness was varied by substance use in order to allow for any one of the following scenarios: (1) missing heroin only; (2) missing cannabis only; (3) missing methamphetamine only; or (4) missing all three drugs simultaneously. The *ampute* package in R was used to generate the missingness [19, 23]. In addition to the three drug use variables (i.e., heroin, methamphetamine, and cannabis use) age and employment status were used to generate missingness in the substance use data. The reason age and employment status were chosen as auxiliary variables is because in the context of this project, these variables serve as a proxy for the specific characteristics of cohorts across which we intend to harmonize data and will help in replicating the most plausible missing data pattern in the context of our work.

#### Data imputation

After the missing data were generated in such a way as to simulate ‘real world’ missing data scenarios that may result during the data harmonization process, various data imputation strategies were used to impute the missing data. The imputation methods used included two different single imputation strategies as well as multiple imputation including: (1) logistic regression; (2) single hot-deck imputation; and (3) multiple imputation with five and twenty imputations. These imputation strategies were chosen since they reflect a range of strategies from simple to complex, both from the technical expertise required to implement as well as the computational resources needed to execute. Specifics of each of the imputation strategies are described below.

Imputation with logistic regression is a single imputation strategy that produces predicted probabilities obtained by regressing the missing variable on other variables [24]. In this case, the specific drug (e.g., methamphetamine) was an outcome variable and age, employment status, and cannabis and/or heroin use served as predictor or auxiliary variables. This strategy is technically relatively simple, preserves relationships among variables involved in the imputation model, and may provide a more informed estimate of the missing value that moves beyond a strategy that ignores other auxiliary variables. Hot-deck imputation is a computationally simple imputation strategy that uses data from an individual in the sample who has similar values on other variables to impute the missing values [11]. Observations imputed are labeled recipients and observations drawn from a pool of matching candidates are labeled donors. For this analysis, donors were matched based on age, employment status, and other substance use information. For example, if a recipient with missing data on methamphetamine was 25 years of old, employed, and reported cannabis use (but no heroin), then all 25 year old, employed participants who reported cannabis use other than the recipient were considered donors and a random observation was taken from this pool and the methamphetamine use status of the selected donor was used for the recipient. Instead of using actual observed values from a donor pool, multiple imputation uses a stochastic logistic regression model to generate n-sets of data – in this analysis n was either five or twenty – given pre-specified auxiliary variables [25, 26]. Each of the resulting datasets were used for analysis and the results are then pooled for inference. The auxiliary variables used were the same as those described above. For example, five predicted data sets were generated for missing cannabis data using a stochastic logistic regression model composed of age, employment status, as well as reported methamphetamine and/or heroin use. Multiple imputation is expected to result in lower bias, however, this strategy is computationally intensive and requires technical expertise that may makes its regular application less practical. Finally, in order to allow for direct comparison between the various imputation strategies, the auxiliary variables were the same in all strategies. The Monte Carlo simulation study from amputation to imputation was conducted using R (version 4.1.1).

#### Evaluation of the performance of imputation strategies

The data amputation and subsequent imputation was repeated 500 times in order to generate a simulated distribution that allowed for calculations to assess the performance of each strategy. We calculated the prevalence estimate resulting from the simulations as an average estimate across the 500 simulations. First, we report prevalence estimates for each of the substances given 10%, 30%, and 50% missingness based on listwise deletion. Listwise deletion, also known as complete case analysis, is the default strategy in most analytic software and provides an estimate of the prevalence and potential magnitude of bias if imputation is not employed. Next, we estimated the magnitude of the potential bias (i.e., mean bias) based on the average difference between the prevalence estimate from the original data and the mean of the prevalence estimate across the 500 simulation replicates. We also provide calculations for the root mean squared error (RMSE) as well as coverage of the 95% confidence interval, which was calculated based on the proportion of times the 95% confidence interval of the estimated summary estimate contained the prevalence estimate from the original data. Each of these statistics were defined as follows:

Mean Bias = $\frac{1}{500}\sum_{i=1}^{500}(\widehat{{p}_{i}}-p)$ where $p$ is the prevalence from the original data of n = 2,389 and $\widehat{{p}_{i}}$ is the estimated prevalence for the *i-th* replication across 500 simulation runs.

Relative Mean Bias = $\frac{\overline{p }-p}{p}\times 100\%$ where $p$ is the prevalence from the original data of n = 2,389 and $\overline{p }=\frac{1}{500}\sum_{i=1}^{500}\widehat{{p}_{i}}$

RMSE = $\sqrt{\frac{1}{500}{\sum_{i=1}^{500}\left(\widehat{{p}_{i}}-p\right)}^{2}}$ where $\widehat{{p}_{i}}$ is the estimated prevalence for the *i-th* replication across 500 simulation runs.

Coverage =$\frac{1}{500}\sum_{i=1}^{500}I\left(p\in C{I}_{i}\right)$ $\times 100\%$ where $C{I}_{i}$ is the *i*-*th* confidence interval.

## Results

### Study population characteristics

At baseline, the average age of participants included in this analysis was 31.2 years, 42.4% identified as African American, 37.9% as Hispanic/Latino, and 13.4% as white (Table 1). Nearly half of the participants reported being unemployed and 35.5% reported unstable housing defined as not having a regular place to stay for at least one night in the past six months. Differences in these characteristics were also noted by HIV status, with HIV-positive participants reporting a higher prevalence of unemployment (55.3%) at baseline as compared to HIV-negative participants (35.6%; p value < 0.01). Across all study visits (n = 2,398), the prevalence of self-reported substance use in the past six months was 51.8% (95% CI: 50.0%-54.0%) for cannabis, 37.5% (95% CI: 35.5%-39.4%) for methamphetamine, and 3.0% (95% CI: 2.3%-3.7%) for heroin. Differences in substance use patterns were noted by HIV status. Participants living with HIV reported higher prevalence of methamphetamine use as compared to participants who were HIV-negative (48.8% vs. 25.3% respectively; p value < 0.01) but lower prevalence of cannabis use (47.5% vs. 56.6% respectively; p value < 0.01).

**Table 1:** Sociodemographic and substance use characteristics of mSTUDY participants (8/2014—06/2019)

	**Total**		**HIV-positive**		**HIV-negative**		*p* value
	**n**	**%**	**n**	**%**	**n**	**%**	
**Baseline characteristics**	**528**	**100.0**	**264**	**100.0**	**264**	**100.0**	–
Age, years (mean, SD)	31.2 (6.8)		33.5(6.5)		29.0 (6.5)		< 0.01
Race/ethnicity							0.61
African American	224	42.4	106	40.2	118	44.7	
Hispanic/Latino	200	37.9	101	38.3	99	37.5	
Other	33	6.3	13	4.9	20	7.6	
White	71	13.4	44	16.7	27	10.2	
Education							0.04
Less than High School	64	12.2	40	15.4	24	9.1	
High School Graduate	189	36.1	94	36.2	95	36.0	
More than High School	271	51.7	126	48.5	145	54.9	
Unemployed	242	45.6	146	55.3	94	35.6	< 0.01
Unstable Housing, past 6 months^a^	190	35.5	91	35.4	92	35.7	0.95
**Total visits with self-reported substance use data**	**2,389**	**100.0**	**1,249**	**100.0**	**1,140**	**100.0**	–
Substance use, past 6 months							
Heroin	75	3.0	33	2.6	42	3.7	0.15
Methamphetamine	897	37.5	609	48.8	288	25.3	< 0.01
Cannabis	1,238	51.8	593	47.5	645	56.6	< 0.01

*Abbreviations*, *SD* Standard deviation

^a^Defined as not having a regular place to stay in the past 6 months

#### Imputation results for a low prevalence substance – the case of heroin

Assuming data were MCAR with 10% missing, estimates without imputation (i.e., listwise deletion) resulted in minimal bias of heroin prevalence estimates when compared to the prevalence in the original data (relative bias + 0.11%)(Table 2). Furthermore, the complete case analysis performed comparable if not better to both single and multiple imputation strategies, even when the missingnes increased to a high of 50%. For instance, the percent relative bias was -0.23% for listwise deletion with 94.2% coverage and 6.03% for multiple imputation (m = 20) with 93.6% coverage of the 95% confidence interval. However, under conditions where data were missing at random multiple imputation provided the least biased estimates while maintaining optimal coverage and lowest RMSE. For instance, assuming 30% missingness MI with 5 imputations resulted in relative bias of 3.95%, with 95.4% coverage, with comparable performance noted for MI with 20 imputations even when missingness increased to 50% (Table 2). Under conditions of MNAR, all strategies performed poorly in providing an estimate for the prevalence of heroin.

**Table 2:** Relative bias, RMSE and coverage probability for heroin use comparing validation data to imputed data

Method	% Missing	Estimate	Mean Bias	% Relative Bias	RMSE	Coverage
*Missing Data Mechanism: MCAR*						
LD	10%	3.0%	0.00003	0.11%	0.0036	94.6%
LR	10%	3.0%	0.00006	0.19%	0.0036	94.6%
HD	10%	3.0%	0.00001	0.04%	0.0036	94.0%
MI (M = 5)	10%	3.0%	0.00034	1.14%	0.0036	94.8%
MI (M = 20)	10%	3.0%	0.00033	1.09%	0.0036	95.0%
LD	30%	3.0%	0.00009	0.29%	0.0042	94.6%
LR	30%	3.0%	0.00010	0.34%	0.0038	92.2%
HD	30%	3.0%	0.00006	0.19%	0.0042	89.6%
MI (M = 5)	30%	3.1%	0.00108	3.60%	0.0040	94.2%
MI (M = 20)	30%	3.1%	0.00105	3.50%	0.0039	94.2%
LD	50%	3.0%	-0.00007	-0.23%	0.0049	94.2%
LR	50%	3.0%	-0.00006	-0.20%	0.0039	90.0%
HD	50%	3.0%	0.00006	0.20%	0.0044	88.6%
MI (M = 5)	50%	3.2%	0.00183	6.09%	0.0045	93.8%
MI (M = 20)	50%	3.2%	0.00181	6.03%	0.0043	93.6%
*Missing Data Mechanism: MAR*						
LD	10%	2.0%	-0.00999	-33.33%	0.0105	14.0%
LR	10%	2.9%	-0.00110	-3.66%	0.0037	91.8%
HD	10%	2.9%	-0.00049	-1.65%	0.0037	92.8%
MI (M = 5)	10%	3.0%	0.00031	1.03%	0.0037	95.0%
MI (M = 20)	10%	3.0%	0.00034	1.12%	0.0037	94.2%
LD	30%	1.4%	-0.01554	-51.84%	0.0158	1.0%
LR	30%	2.7%	-0.00256	-8.55%	0.0044	83.4%
HD	30%	2.9%	-0.00132	-4.40%	0.0043	87.2%
MI (M = 5)	30%	3.1%	0.00118	3.95%	0.0042	95.4%
MI (M = 20)	30%	3.1%	0.00121	4.02%	0.0041	95.2%
LD	50%	1.1%	-0.01871	-62.41%	0.0189	0.0%
LR	50%	2.7%	-0.00337	-11.25%	0.0051	75.6%
HD	50%	2.8%	-0.00151	-5.03%	0.0048	82.8%
MI (M = 5)	50%	3.2%	0.00201	6.69%	0.0048	93.6%
MI (M = 20)	50%	3.2%	0.00205	6.83%	0.0046	93.8%
*Missing Data Mechanism: MNAR*						
LD	10%	1.6%	-0.01361	-45.41%	0.0139	0.8%
LR	10%	1.8%	-0.01181	-39.41%	0.0122	3.4%
HD	10%	1.9%	-0.01131	-37.73%	0.0117	7.0%
MI (M = 5)	10%	1.9%	-0.01101	-36.74%	0.0114	11.4%
MI (M = 20)	10%	1.9%	-0.01099	-36.67%	0.0114	10.8%
LD	30%	1.2%	-0.01799	-60.03%	0.0182	0.0%
LR	30%	1.8%	-0.01192	-39.77%	0.0123	3.2%
HD	30%	1.9%	-0.01127	-37.59%	0.0117	6.8%
MI (M = 5)	30%	2.0%	-0.01016	-33.91%	0.0106	20.6%
MI (M = 20)	30%	2.0%	-0.01023	-34.13%	0.0106	18.6%
LD	50%	1.0%	-0.02032	-67.82%	0.0205	0.0%
LR	50%	2.0%	-0.00994	-33.15%	0.0105	13.8%
HD	50%	2.1%	-0.00934	-31.16%	0.0101	22.8%
MI (M = 5)	50%	2.3%	-0.00687	-22.91%	0.0077	69.8%
MI (M = 20)	50%	2.3%	-0.00688	-22.97%	0.0077	65.8%

*Abbreviations*, *LD* Listwise deletion, *LR* Logistic regression, *HD* Hot-deck, *MI* Multiple imputation, *MCAR* Missing completely at random, *MAR* Missing at random, *MNAR* Missing not at random, *RMSE* Root mean square error

#### Imputation results for a medium prevalence substance – the case of methamphetamine

Assuming an MCAR missing data mechanism, the amount of bias resulting from missingness in methamphetamine prevalence estimates was low. For instance, the prevalence of methamphetamine use in the original data was 37.5% and without imputation (i.e., listwise deletion) prevalence estimates ranged from 37.4% (coverage: 95.6%; RMSE: 0.0105) under conditions with 10% missingness, 37.5% (coverage: 95.2%; RMSE: 0.0117) under conditions of 30% missingness, and 37.4% (coverage: 94.6%; RMSE: 0.0148) under conditions with 50% of the data missing (Table 3). Additionally, under conditions of MCAR all imputation strategies performed comparably. However, differences were noted across the various imputation strategies when the missing data mechanism under consideration was MAR. Lack of imputation with MAR data resulted in increasing levels of bias as the levels of missingness increased. Of note, with regards to multiple imputation, similar results were obtained from imputations with five and twenty data sets. For instance methamphetamine prevalence was estimated at 37.5% for MI with five imputations and 30% missingness (relative bias: 0.12%; coverage: 95.6%; RMSE: 0.0110) as compared to 37.5% for MI with twenty imputations (relative bias: 0.14%; coverage: 96.0%; RMSE: 0.0109) (Table 3).

**Table 3:** Relative bias percentage, RMSE and coverage probability for methamphetamine use comparing validation data to imputed data

Method	% Missing	Estimate	Mean Bias	% Relative Bias	RMSE	Coverage
*Missing Data Mechanism: MCAR*						
LD	10%	37.4%	-0.00055	-0.15%	0.0105	95.6%
LR	10%	37.5%	-0.00031	-0.08%	0.0105	94.2%
HD	10%	37.4%	-0.00035	-0.09%	0.0108	94.0%
MI (M = 5)	10%	37.5%	-0.00013	-0.03%	0.0115	95.0%
MI (M = 20)	10%	37.5%	-0.00013	-0.04%	0.0128	95.2%
LD	30%	37.5%	-0.00003	-0.01%	0.0117	95.2%
LR	30%	37.4%	-0.00035	-0.09%	0.0110	93.2%
HD	30%	37.5%	-0.00017	-0.04%	0.0115	92.2%
MI (M = 5)	30%	37.5%	0.00040	0.11%	0.0115	95.4%
MI (M = 20)	30%	37.5%	0.00021	0.06%	0.0128	95.4%
LD	50%	37.4%	-0.00064	-0.17%	0.0148	94.6%
LR	50%	37.5%	-0.00005	-0.01%	0.0117	89.4%
HD	50%	37.5%	0.00002	0.01%	0.0128	87.8%
MI (M = 5)	50%	37.6%	0.00071	0.19%	0.0115	95.2%
MI (M = 20)	50%	37.6%	0.00071	0.19%	0.0128	94.2%
*Missing Data Mechanism: MAR*						
LD	10%	34.9%	-0.02540	-6.78%	0.0276	30.8%
LR	10%	36.7%	-0.00818	-2.18%	0.0133	83.8%
HD	10%	37.3%	-0.00216	-0.58%	0.0108	93.0%
MI (M = 5)	10%	37.5%	0.00034	0.09%	0.0104	94.6%
MI (M = 20)	10%	37.5%	0.00036	0.10%	0.0103	94.4%
LD	30%	30.8%	-0.06707	-17.89%	0.0680	0.0%
LR	30%	35.7%	-0.01803	-4.81%	0.0210	56.0%
HD	30%	37.1%	-0.00338	-0.90%	0.0121	88.6%
MI (M = 5)	30%	37.5%	0.00044	0.12%	0.0110	95.6%
MI (M = 20)	30%	37.5%	0.00054	0.14%	0.0109	96.0%
LD	50%	26.9%	-0.10551	-28.15%	0.1063	0.0%
LR	50%	35.2%	-0.02289	-6.11%	0.0257	37.4%
HD	50%	37.1%	-0.00364	-0.97%	0.0134	86.6%
MI (M = 5)	50%	37.6%	0.00082	0.22%	0.0120	94.4%
MI (M = 20)	50%	37.6%	0.00076	0.20%	0.0118	93.0%
*Missing Data Mechanism: MNAR*						
LD	10%	35.0%	-0.02520	-6.72%	0.0274	32.6%
LR	10%	35.5%	-0.01959	-5.22%	0.0223	50.2%
HD	10%	35.8%	-0.01650	-4.40%	0.0197	61.6%
MI (M = 5)	10%	35.8%	-0.01651	-4.41%	0.0196	61.4%
MI (M = 20)	10%	35.8%	-0.01651	-4.40%	0.0196	62.2%
LD	30%	35.0%	-0.02520	-6.72%	0.0274	32.6%
LR	30%	35.5%	-0.01959	-5.22%	0.0223	50.2%
HD	30%	33.5%	-0.04014	-10.71%	0.0417	3.0%
MI (M = 5)	30%	35.8%	-0.01651	-4.41%	0.0196	61.4%
MI (M = 20)	30%	33.5%	-0.03958	-10.56%	0.0410	3.6%
LD	50%	27.9%	-0.09619	-25.66%	0.0971	0.0%
LR	50%	31.6%	-0.05870	-15.66%	0.0598	0.0%
HD	50%	32.4%	-0.05061	-13.50%	0.0521	0.8%
MI (M = 5)	50%	32.6%	-0.04917	-13.12%	0.0505	2.2%
MI (M = 20)	50%	32.6%	-0.04914	-13.11%	0.0504	1.6%

*Abbreviations*, *LD* Listwise deletion, *LR* Logistic regression, *HD* Hot-deck, *MI* Multiple imputation, *MCAR* Missing completely at random, *MAR* Missing at random, *MNAR* Missing not at random, *RMSE* Root mean square error

#### Imputation results for a high prevalence substance – the case of cannabis

Comparable to the scenarios with low and medium prevalence outcomes, both single and multiple imputation strategies with lower levels of missingness with an MCAR missing data mechanism performed well (Table 4). Additionally, none of the strategies were effective under circumstances where data were MNAR. For all levels of missingness and assuming data were MAR, multiple imputation outperformed all strategies with both five and twenty imputed data sets resulting in comparable outcomes. For instance, with 50% missingnes, MI with five and twenty data sets resulted in a prevalence estimate of 52%, minimal bias (0.06% and 0.05%, respectively) and otherwise comparable in terms of coverage (95.5% and 94.4%, respectively) and RMSE (0.0122 in both cases).

**Table 4:** Relative bias percentage, RMSE and coverage probability for cannabis use comparing validation data to imputed data

Method	% Missing	Estimate	Mean Bias	% Relative Bias	RMSE	Coverage
*Missing Data Mechanism: MCAR*						
LD	10%	52.0%	0.00046	0.09%	0.0299	95.6%
LR	10%	52.0%	0.00042	0.08%	0.0285	95.8%
HD	10%	52.0%	0.00033	0.06%	0.0253	94.2%
MI (M = 5)	10%	52.0%	0.00056	0.11%	0.0328	95.6%
MI (M = 20)	10%	52.0%	0.00050	0.10%	0.0311	96.2%
LD	30%	52.0%	0.00021	0.04%	0.0201	94.8%
LR	30%	52.0%	0.00029	0.06%	0.0238	93.0%
HD	30%	52.0%	0.00034	0.07%	0.0256	92.0%
MI (M = 5)	30%	52.0%	0.00032	0.06%	0.0247	95.8%
MI (M = 20)	30%	52.0%	0.00036	0.07%	0.0262	95.6%
LD	50%	52.0%	0.00070	0.14%	0.0368	94.2%
LR	50%	52.0%	0.00072	0.14%	0.0373	91.0%
HD	50%	52.0%	0.00057	0.11%	0.0332	87.4%
MI (M = 5)	50%	52.0%	0.00064	0.12%	0.0351	95.4%
MI (M = 20)	50%	52.0%	0.00079	0.15%	0.0389	95.0%
*Missing Data Mechanism: MAR*						
LD	10%	51.0%	-0.00991	-1.91%	0.0145	85.4%
LR	10%	52.0%	0.00032	0.06%	0.0102	95.8%
HD	10%	51.8%	-0.00134	-0.26%	0.0107	93.6%
MI (M = 5)	10%	52.0%	0.00027	0.05%	0.0101	96.2%
MI (M = 20)	10%	52.0%	0.00034	0.07%	0.0102	96.2%
LD	30%	49.5%	-0.02443	-4.70%	0.0273	48.8%
LR	30%	52.2%	0.00207	0.40%	0.0112	93.2%
HD	30%	51.7%	-0.00241	-0.46%	0.0118	91.2%
MI (M = 5)	30%	52.0%	0.00054	0.10%	0.0110	95.8%
MI (M = 20)	30%	52.0%	0.00038	0.07%	0.0111	95.0%
LD	50%	48.0%	-0.03969	-7.64%	0.0421	21.0%
LR	50%	52.3%	0.00340	0.65%	0.0124	90.4%
HD	50%	51.7%	-0.00280	-0.54%	0.0137	86.0%
MI (M = 5)	50%	52.0%	0.00029	0.06%	0.0122	95.0%
MI (M = 20)	50%	52.0%	0.00028	0.05%	0.0122	94.4%
*Missing Data Mechanism: MNAR*						
LD	10%	49.9%	-0.02029	-3.90%	0.0230	53.8%
LR	10%	50.4%	-0.01557	-3.00%	0.0188	69.0%
HD	10%	50.5%	-0.01480	-2.85%	0.0183	70.4%
MI (M = 5)	10%	50.5%	-0.01495	-2.88%	0.0183	72.6%
MI (M = 20)	10%	50.5%	0.47888	92.16%	0.0183	72.2%
LD	30%	46.1%	-0.05821	-11.20%	0.0596	0.0%
LR	30%	47.7%	-0.04286	-8.25%	0.0444	1.8%
HD	30%	47.9%	-0.04112	-7.91%	0.0429	3.6%
MI (M = 5)	30%	47.9%	-0.04078	-7.85%	0.0423	4.0%
MI (M = 20)	30%	-1.5%	-0.04075	-7.84%	0.0423	4.0%
LD	50%	42.2%	-0.09781	-18.82%	0.0988	0.0%
LR	50%	45.9%	-0.06050	-11.64%	0.0616	0.0%
HD	50%	46.1%	-0.05828	-11.22%	0.0597	0.0%
MI (M = 5)	50%	46.3%	-0.05702	-10.97%	0.0582	0.0%
MI (M = 20)	50%	0.0%	0.00013	0.02%	0.0582	0.0%

*Abbreviations LD* Listwise deletion, *LR* Logistic regression, *HD* Hot-deck, *MI* Multiple imputation, *MCAR* Missing completely at random, *MAR* Missing at random, *MNAR* Missing not at random, *RMSE* Root mean square error

## Discussion

We evaluated the performance of different imputation strategies used to address missingness in key variables that thwart efforts to harmonize data collected as part of HIV-cohort studies. Our findings suggest that while multiple imputation is an effective tool for re-creating unbiased prevalence rates of substance use under MAR, single imputation strategies may also be effective if the missing data mechanism is MCAR. Furthermore, we demonstrate that when the missing data mechanism is MAR (which is the likely case in these harmonization efforts), ignoring the missingness can result in underestimation of the prevalence estimates and that single imputation strategies are ineffectual in correcting this bias, especially in cases where the prevalence of the outcome is low. Finally, we demonstrate that none of the imputation strategies are effective if missingness is not at random (i.e., MNAR).

Most of the imputation strategies resulted in a significant improvement in estimates of substance use data when compared to estimates relying on complete case data, which was especially true in the case of a ‘rare’ outcome such as heroin use. However, MI performed the most favorably (assuming the data were not MNAR) regardless of the prevalence of the outcome or the level of missingness. Missing data resulting from combining data across cohorts more plausibly resembles an MAR data mechanism given that missing substance use data is related to other observed data, namely cohort characteristics such as age and HIV status. We used plausible proxy cohort characteristics such as age, employment status, and other substance use variables to inform the imputation process, which likely results in both gains in efficiency and reductions in bias [27]. The finding that five imputation data sets performed nearly as well as multiple imputation with twenty data sets addresses the practical decision of how many imputations are needed. While some of the original work in this area suggests that between two and ten imputations are sufficient, others have suggested that more than ten and up to 100 imputations may be needed if the fraction of missing information is large [25, 27–29]. Our findings suggest that efficiency can be maintained with smaller imputed data sets, even under circumstances where up to 50% of the data are being imputed.

We recognize that if MI works in all scenarios, then having to make a decision to use another imputation strategy may be unnecessary. However, because the goal is to implement these strategies in the context of cross protocol analyses requiring harmonization across multiple cohorts with thousands of observations this can create a number of practical issues. First, researchers conducting cross-cohort analyses will need to have the technical expertise needed to analyze MI data sets which requires running statistical models on each of the imputed data sets and then pooling the resulting model parameter estimates. So, assuming twenty imputations are done, then this step will result in running statistical models on twenty sets of data, obtaining the twenty resulting parameter estimates, which then are pooled into a single pooled estimate. Furthermore, practical considerations also come in to play given the potentially large sample sizes along with imputations creating up to twenty data sets. The potential impact on computer processing power or statistical programming requirements may make such analyses difficult for many investigators.

The findings of this study should be interpreted in light of some of the limitations. We utilized a cross sectional view of the data and did not give consideration to a monotone missing data pattern resulting from loss to follow-up given that imputation for missing data resulting from loss to follow-up was not the goal of this study [30]. Assessment of substance use was based on self-report and any response bias introduced as part of the data collection are not corrected for as part of these analyses. However, our use of computer assisted self-interview (CASI) may have helped to improve the validity of the self-reported information [31, 32]. Nonetheless, our imputations were strengthened by the use of auxiliary variables. In particular our use of other substance use data is particularly relevant in that it will allow us to use participants’ reported substance use patterns to inform our imputations. A restrictive strategy that does not make use of auxiliary variables is less informative and as others have noted the more inclusive strategy reduces the chance of inadvertent omission of important causes of missing data with resulting gains in efficiency and reduction in bias [27]. The only cost here is the availability of data for the selected auxiliary variables.

## Conclusions

In conclusion, our analyses reveal that while MI with as few as five imputations provided the best performance across a range of conditions, single imputation using logistic regression or the hot-deck method for substance use data was a robust strategy under certain circumstances where the data are assumed to be missing completely at random or the level of missingness is low. Ignoring the missingness will bias our results and limit the utility of combining data collected as part of the individual studies. While we can uniformly advocate the use of MI, we also suggest that under certain circumstances single imputation may be a viable option given its relatively low bias and ease of implementation.

## Data Availability

The datasets used and/or analyzed during the current study are available from the corresponding author on reasonable request.
